# Virus detection by short read high throughput sequencing in a high virus low cellular background

**DOI:** 10.1038/s41541-025-01104-1

**Published:** 2025-03-29

**Authors:** Pei-Ju Chin, Christophe Lambert, Pascale Beurdelay, Robert L. Charlebois, Anne-Sophie Colinet, Marc Eloit, Shanaz Gilchrist, Maria Gilleece, Matthew Hess, Andreas Leimbach, Tom J. B. de Man, Olivier Vandeputte, Dawid Walas, Weihong Wang, Arifa S. Khan

**Affiliations:** 1 Office of Vaccines Research and Review, Center for Biologics Evaluation and Review, U.S. Food and Drug Administration, Silver Spring, MD 20993 USA; 2 GSK, Rue de l’Institut 89, Rixensart, Belgium; 3 PathoQuest, 11 rue Watt, Paris, France; 4 Sanofi, 1755 Steeles Avenue West, Toronto, ON Canada; 5 Biogen 5000 Davis Drive, Durham, NC USA; 6 Eurofins Genomics Europe Pharma and Diagnostics Products & Services Sanger/PCR GmbH, Konstanz, Germany; 7 MilliporeSigma, Rockville, MD USA; 8 Eurofins Lancaster Laboratories, Inc, Lancaster, PA USA; 9 Present Address: Depixus, 3-5 Imp. Reille, Paris, France; 10 Present Address: Institut de Recherche et Development Servier, 91190 Gif-sur-Yvette, France and Department of Physiology, Institute of Medical Sciences, University of Opole, Opole, Poland

**Keywords:** Virology, Biologics

## Abstract

The safety of all biological products includes demonstrating the absence of adventitious viruses by testing various types of samples at different stages of the manufacturing process. Seven laboratories evaluated short-read high-throughput sequencing (HTS) for sensitivity and breadth of adventitious virus detection using viruses with distinct physicochemical and genome properties. These five viruses are currently designated as CBER NGS Virus Reagents and include: Epstein-Barr virus (EBV; or human herpes virus 4), feline leukemia virus (FeLV), respiratory syncytial virus (RSV), mammalian orthoreovirus type 1 (Reo1), and porcine circovirus type 1 (PCV1). To evaluate adventitious virus detection in a biological material with a high production virus titer and low cellular background, the 5 viruses were mixed and different copies of the viral genomes spiked into 1 – 5 × 10^9^ genome copies per mL (GC/mL) of purified adenovirus 5. Independent protocols were used by each laboratory for the entire HTS workflow. All laboratories detected 10^4^ GC/mL of the five viruses by both targeted and non-targeted bioinformatic analyses. Additionally, the limit of detection of squirrel monkey retrovirus and porcine endogenous retrovirus, which pre-existed in EBV and PCV1 virus stocks, respectively, was evaluated. The five laboratories that tested 10^3^ GC/mL, detected all 5 viruses with the targeted analysis, and Reo1 and EBV with the non-targeted analysis. It was noted that some laboratories achieved a better sensitivity for detection of the five viruses ( ≤10^2^ GC/mL). This study presents an approach for HTS validation for viral safety testing of vaccines and other biologics using a panel of reference viruses. The results highlight that optimization of steps in the HTS workflow can improve the limit of detection for adventitious viruses.

## Introduction

High-throughput sequencing (HTS), also referred to as next-generation sequencing (NGS) or massively parallel sequencing (MPS), is an advanced nucleic acid-based technology that can detect viruses, without prior sequence knowledge. HTS has contributed significantly to virus discovery from clinical, environmental, and biological materials resulting in expansion of the known virome^1–7^. The commercial availability of different HTS platforms along with gain of experience in understanding the strengths and limitations of the technologies have resulted in the introduction and increasing use of HTS for adventitious virus detection in biologics^8^. Although some aspects of HTS are still maturing, it has been demonstrated that HTS can address the limitations of the currently recommended in vivo and in vitro assays for broad adventitious virus detection in viral vaccines and other biological products^6,7^. With the publication of the ICH Q5A(R2) guideline^9^ and supporting data^10–12^, HTS is recognized as an alternative method for supplementing or replacing the conventional adventitious virus tests to enhance viral safety of biologics^9^.

While the use of HTS for adventitious virus detection in biologics is increasing to facilitate rapid product development, efforts are still needed for optimizing protocols and generating reference materials for HTS validation and routine applications. HTS workflows are complex with various steps involved in the upstream sample processing, different sequencing technologies and platforms, and customized computational pipelines used in downstream bioinformatics analyses. The entire workflow needs to be evaluated, end-to-end as a whole or each step individually, for demonstrating the capability of HTS for virus detection. Upstream sample processing includes using different kits and/or protocols for nucleic acid extraction, cDNA synthesis, and library preparation^13^. Downstream bioinformatics analyses require assessment of tools and parameters used in the HTS data analysis pipelines^14^, and a comprehensive viral database^15^ to facilitate broad virus detection including known and novel sequences. The increased data output along with the continuing increase in the size of reference databases due to the recent addition of the multitude of SARS-CoV-2 genomes^16,17^ has further challenged the computational pipelines for HTS bioinformatics analyses. Furthermore, HTS instruments are diverse with different outputs based on number and length of reads^18^ generated by using short-read or long-read technologies.

To understand some of the challenges of using HTS for adventitious virus detection in biologics, this study has focused on evaluating the sensitivity and breadth of virus detection in a sample with a high-virus titer and low-cellular background, mimicking a purified viral vaccine seed or a viral vector preparation using the Illumina short-read technology. Other types of materials tested during the manufacturing process can have a higher level of host cell nucleic acids that would need further optimization of HTS for virus detection; for example, cells used for production and the unprocessed bulk harvest material, which is an intermediate for the final product, The viruses used represented diverse physical, chemical, and genome properties for performance evaluation of the different steps involved in HTS. Seven laboratories conducted a virus spiking study, as part of the Advanced Virus Detection Technologies Working Group (AVDTWG, previously named Interest Group AVDTIG)^19^, which consisted of laboratories involved in product manufacturing, testing, or regulating biological products. The five CBER NGS Virus Reagents [i.e., porcine circovirus type 1 (PCV1), human herpes virus 4 also known as Epstein-Barr virus (EBV), feline leukemia virus (FeLV), human respiratory syncytial virus (RSV), and mammalian orthoreovirus type 1 (Reo1)^20,21^] were used for spiking into high-titer purified adenovirus 5 (Ad5). While the same virus stocks were used by all of the laboratories for preparing the spiked samples, independent HTS workflows were used by each participant. This collaborative study determined the analytical sensitivity and breadth of detection of the five reference viruses at varying spike levels based on genome copies per mL (GC/mL) in high-titer Ad5. Additionally, the detection limit of squirrel monkey retrovirus (SMRV) and porcine endogenous retrovirus (PERV), which were known to be present in the EBV and PCV1 virus stocks, respectively, due to their presence in the production cell lines, was calculated based on their concentrations in the reference virus stocks. The detection of the viruses representing a wide range of physicochemical and genomic properties of diverse virus families demonstrated an approach for using reference standards for qualification and validation of the HTS workflow for broad adventitious virus detection in biologics. Additionally, the results of the study indicated an expected limit of detection for adventitious viruses in a similar matrix using short-read HTS for viral safety testing of biologics.

## Results

HTS data analysis was performed using targeted bioinformatic analysis for detection of the five spiked viruses (referred to as reference viruses) in the spiked sample. In order to mimic HTS applications in biologics, non-targeted bioinformatic analysis was also performed for broader virus detection, including known viral and related sequences as well as unrelated viral sequences, which could be due to reagents or other sources. The overall HTS results from individual laboratories are shown in the Supplementary Table 1. The summary results and comparison of HTS virus detection using targeted and non-targeted bioinformatics analyses are described in this section.

### HTS targeted analysis for detection of the expected viruses used in the spiking study

In the targeted analysis, HTS reads were mapped to the reference genomes of the viruses used in the spiking study to determine the analytical sensitivity of virus detection. The virus spike level (SL) used in the study ranged from 1 × 10^1^ to 5 × 10^6^ GC/mL of the spiked viruses in the background of 1 × 10^9^ to 5 × 10^9^ GC/mL of Ad5 (designated as SL-1 through SL-6, respectively, in Table 1). Although all the different virus spike levels were not tested by each laboratory, SL-4 was included by all the laboratories for a comparison of virus detection using different sample preparation protocols, sequencing platforms, and bioinformatics pipelines. Furthermore, the level of detection of PERV and SMRV, which were extraneous viruses pre-existing in the PCV1 and EBV virus stocks, respectively (described in Materials and Methods), was also evaluated based on their known concentration (GC/mL) in the spiked reference virus stocks. The overall study results of virus detection are shown in Table 1.

**Table 1:** Targeted HTS analysis showing patterns of virus detection at different virus spiked levels for each participating laboratory

Spiked level of reference virus genome (SL)^a^	Lab ID	Reference Virus					Additional Virus	
		PCV1	EBV	FeLV	RSV	Reo1	PERV^b^	SMRV^c^
SL-6	2, 3	+	+	+	+	+	+ ( ≈ 4)	+ ( ≈ 7)
SL-5	5	+	+	+	+	+	+ ( ≈ 3)	+ ( ≈ 6)
SL-4	1–3, 5–7	+	+	+	+	+	+ ( ≈ 2)	+ ( ≈ 5)
	4	+	+	+	+	+	− ( ≈ 2)	+ ( ≈ 5)
SL-3	1, 3, 5	+	+	+	+	+	+ ( ≈ 1)	+ ( ≈ 4)
	4, 6	+	+	+	+	+	− ( ≈ 1)	+ ( ≈ 4)
SL-2	2	+	+	−	+	−	− ( ≈ 0)	+ ( ≈ 3)
	3	−	+	+	+	−	+ ( ≈ 0)	+ ( ≈ 3)
	4	−	+	−	−	−	− ( ≈ 0)	+ ( ≈ 3)
	5	+	+	+	+	+	− ( ≈ 0)	+ ( ≈ 3)
	7	+	+	+	−	+	+ ( ≈ 0)	+ ( ≈ 3)
SL-1	5	−	+	+	−	+	− (≈ -1)	+ ( ≈ 2)

^a^The five reference viruses were spiked together at 1 × 10^x^ GC/mL into 1 × 10^9^ GC/mL of Ad5, except that two laboratories spiked the final virus genome copies into the final volume used for their extractions so the reference viruses and Ad5 GC/mL were calculated as 2.5 × 10^x^ for Laboratory 3 and 5 × 10^x^ for Laboratory 4. Exponent × indicates the spike level. The same applies to the additional viruses. For simplicity, the spiked viruses are indicated as spike level (SL) 2 to 6 with GC/mL ranging from SL-2 = 1 × 10^2^ to 5 × 10^2^ to SL-6 = 1 × 10^6^ to 2.5 × 10^6^, respectively. SL-1 (tested only by one laboratory) = 1 × 10^1^.

^b^The concentration of PERV in SL-4 (tested by all laboratories) was 1.4 × 10^2^ GC/mL, except for Laboratories 3 and 4, where PERV was 3.5 × 10^2^ GC/mL and 7 × 10^2^ GC/mL, respectively (as explained in footnote a). The spiked level of PERV (GC/mL) corresponding to that of the reference viruses is indicated in the parenthesis as the exponent of base 10.

^c^SMRV corresponding to the reference virus SL-6 was 3.0 × 10^7^ GC/mL, which was calculated for Laboratories 3 and 4 as 7.5 × 10^7^ GC/mL and 1.5 × 10^8^ GC/mL, respectively. The spiked level of SMRV (GC/mL) corresponding to that of the reference viruses is indicated in the parenthesis as the exponent of base 10.

The results show that the five reference viruses were detected at SL-4 by all laboratories and the five laboratories that tested SL-3 could detect all viruses; however, at SL-2 the results were variable: laboratories 5 detected all five viruses, laboratory 7 detected four viruses (but not RSV), laboratories 2 and 3 could detect three viruses (both detected EBV and RSV, could not detect Reo1, but they differed in the detection of PCV1 and FeLV), and Laboratory 4 only detected EBV. Laboratory 5 was the only lab who analyzed SL-1 and detected EBV, FeLV and Reo1.

Regarding detection of the additional retroviruses that were present in the EBV and PCV1 stocks, all laboratories that tested SL-2, could detect SMRV present at 1 to 5 × 10^3^ GC/mL in this spike level of the reference virus genomes and laboratory 5 could also detect SMRV in the SL-1. Detection of PERV was variable at spiking levels below SL-5, which contained 1.4 × 10^3^ GC/mL of PERV sequences. The results of virus detected (+) or not detected (-) shown in Table 1 are based on laboratories’ criteria described for targeted HTS in Materials and Methods.

### Non-targeted HTS bioinformatic analyses for broad virus detection

To mimic a real-case scenario for broad adventitious virus testing of a biological product, non-targeted analysis was performed by aligning the HTS reads to the Reference Viral Database (RVDB; see Material and Methods). This provided the opportunity for detection of the known spiked virus genomes and other related sequences as well as any other unrelated sequences that may be introduced due to the reagents or facilities used performing the HTS workflow or present in the reagents used to prepare the virus stocks or generate the test samples. Each laboratory performed the non-targeted analysis using their own bioinformatics pipeline.

The results in Table 2 show that all spiked viruses were detected at SL-4 by all laboratories, which was the lowest common level tested. Laboratory 5 detected all the viruses at SL-3 and SL-2 and four of the five viruses at SL-1; laboratory 6 detected all of the spiked viruses at SL-3, which was their lowest level tested. Virus detection below 10^4^ genome copies varied for the other laboratories: in general, three of the five viruses were detected and there was no clear pattern of the viruses detected. Overall, EBV was detected by all of the groups at all SLs tested, and detection of FeLV was the lowest.

**Table 2:** Non-targeted HTS analysis showing patterns of virus detection at different spiked levels for each participating laboratory

Spiked Level of Reference Virus Genome (SL)^a^	Lab ID	Reference Virus					Additional Virus	
		PCV1	EBV	FeLV	RSV	Reo1	PERV^b^	SMRV^c^
SL-6	2, 3	+	+	+	+	+	+ ( ≈ 4)	+ ( ≈ 7)
SL-5	5	+	+	+	+	+	+ ( ≈ 3)	+( ≈ 6)
SL-4	1, 3, 5, 6, 7	+	+	+	+	+	+ ( ≈ 2)	+( ≈ 5)
	2, 4	+	+	+	+	+	− ( ≈ 2)	+( ≈ 5)
SL-3	1	+	+	−	+	+	+ ( ≈ 1)	+( ≈ 4)
	3	−	+	+	−	+	+ ( ≈ 1)	+( ≈ 4)
	4	+	+	−	+	+	− ( ≈ 1)	+( ≈ 4)
	5, 6	+	+	+	+	+	− ( ≈ 1)	+( ≈ 4)
SL-2	2	+	+	−	+	−	− ( ≈ 0)	+( ≈ 3)
	3, 4	−	+	−	−	−	− ( ≈ 0)	+( ≈ 3)
	5	+	+	+	+	+	− ( ≈ 0)	+( ≈ 3)
	7	+	+	−	−	+	− ( ≈ 0)	+( ≈ 3)
SL-1	5	−	+	+	+	+	− (≈ -1)	+( ≈ 2)

^a,b,c^ Refer to footnotes in Table 1.

Detected (+) or not detected (−) based on laboratories’ criteria as described for non-targeted HTS in Materials and Methods.

The detection level of the additional retroviruses by non-targeted analysis was similar to the targeted analysis: all of the laboratories that tested SL-2 could detect SMRV at 3–15 × 10^3^ genome copies. Six of the seven laboratories detected PERV at 1.4–7 × 10^2^ genome copies in SL-4 in the targeted analysis, and five of the seven laboratories detected PERV in the non-targeted analysis (Laboratories 2 and 4 did not).

### HTS detection of unexpected virus sequences by non-targeted bioinformatic analysis

In addition to detecting the expected spiked virus sequences and related viral sequences based on sequence identity, some unexpected viral sequences were also detected by each laboratory (results of spiked level SL-4 are shown in Supplementary Table 1). The number and taxonomic identity of viral sequences varied across laboratories, which was expected since different sample preparation protocols, sequencing platforms, bioinformatics strategies, and criteria for reporting a positive hit were used. Detailed analysis of one spiked level (SL-4) was done to compare the unexpected viral sequences detected by the different laboratories (summary results are shown in Table 3). Most were due to crossmatch with viral sequences expected to be present in the host cells that were used for virus production or with viruses related to the reference viruses from another host species. Furthermore, sequences of ungulate copiparvovirus 5 [bovine serum associated, bovine bosavirus^5^] was detected by laboratories 5 and 7 (for other tested spiked levels, Laboratories 2 and 3 detected it at SL-6 and Laboratory 5 also detected it at SL-5, data not shown). The presence of bosavirus sequence was confirmed by targeted analysis in the PCV1, EBV and Reo1 virus stocks (Chin, unpublished data). The other sequences that were detected by the non-targeted HTS analysis by each laboratory are provided in the Supplementary Table 2.

**Table 3:** HTS detection of additional viral sequences by non-targeted analysis^a^

Related to background virus Adenovirus 5	Laboratory ID
Pan troglodytes adenovirus	1
Unidentified adenovirus	6
Untyped Human adenovirus	6
Untyped Human mastadenovirus	6
***Related to spiked viruses FeLV or SMRV***	
Baboon endogenous virus	1, 7
Primate T-lymphotropic virus 1	3
Macaca mulatta type C retrovirus	1, 3
Mason-Pfizer monkey virus	1, 3, 5, 6
Murine AIDS virus-related provirus	6
Murine leukemia virus	3, 6, 7
Murine leukemia-related retroviruses	6
Mus musculus mobilized endogenous polytropic provirus	3
Simian retrovirus 8	7
**Related to viral sequences present in host cell used for virus propagation**	
Alphapapillomavirus 12	1
Alphapapillomavirus 7	1, 5
Human papillomavirus	6, 7
Human polyomavirus 5	7
**Due to ERVs in host cell DNA**	
Chlorocebus aethiops isolate CA_NCAI14 retrotransposons HERVIP10F_ints, LTR8, and HERVIP10F_int, complete sequence	1
Human endogenous retrovirus	1, 6
Human endogenous retrovirus H	3
Human endogenous retrovirus K	1, 3, 6
**Due to bovine serum**	
Ungulate copiparvovirus 5	5, 7
**Other viral sequences**	
Adeno-associated dependoparvovirus B	1
Autographa californica multiple nucleopolyhedrovirus	7
Blacklegged tick picorna-like virus 2	1
Bovine alphaherpesvirus 5	6
CRESS virus sp.	5
CRESS virus sp. ctin15	5
Crimean-Congo hemorrhagic fever orthonairovirus	6
Guanarito mammarenavirus	1, 3
Human alphaherpesvirus 1	6
Hepatitis B virus	1
Hubei tombus-like virus 8	4
Human immunodeficiency virus 1	1, 4
Lassa mammarenavirus	3
Norway luteo-like virus 4	5
Paramecium bursaria Chlorella virus A1	5
Paramecium bursaria Chlorella virus CVB-1	5
Pepper mild mottle virus	3
Saccharomyces cerevisiae killer virus M1	1
Semliki Forest virus	1
Shamonda orthobunyavirus	3
Sindbis virus	3
Uncultured virus	3, 5
Vaccinia virus	2

^a^Includes all signals reported by the indicated laboratories in SL-4 sample.

Independent HTS bioinformatics pipelines were used by each laboratory for the NGS data analysis. Some also included steps for confirmation of a positive hit as being virus-specific, whereas others did not. Therefore, the results in Table 3 may be an over-representation of viral hits and further investigations are needed to identify whether the results are true viral signals. A known example is the detection of HIV-1 (accession number MT154980.1) by laboratories 1 and 4, which was due to the nonviral sequences present in this HIV-1 sequence. This accession number matched a human chromosome by blastn analysis using the NCBI nr/nt database and was identified as “mitochondria/rRNA/ubiquitin” in the annotation of viral and nonviral sequences on the RVDB website^22^. Therefore, additional analyses would be needed to determine whether the signals are associated with a virus contamination or due to cross-reactivity with nonviral sequences.

### Linearity and detection limits of reference virus sequences

**Fig. 1:**
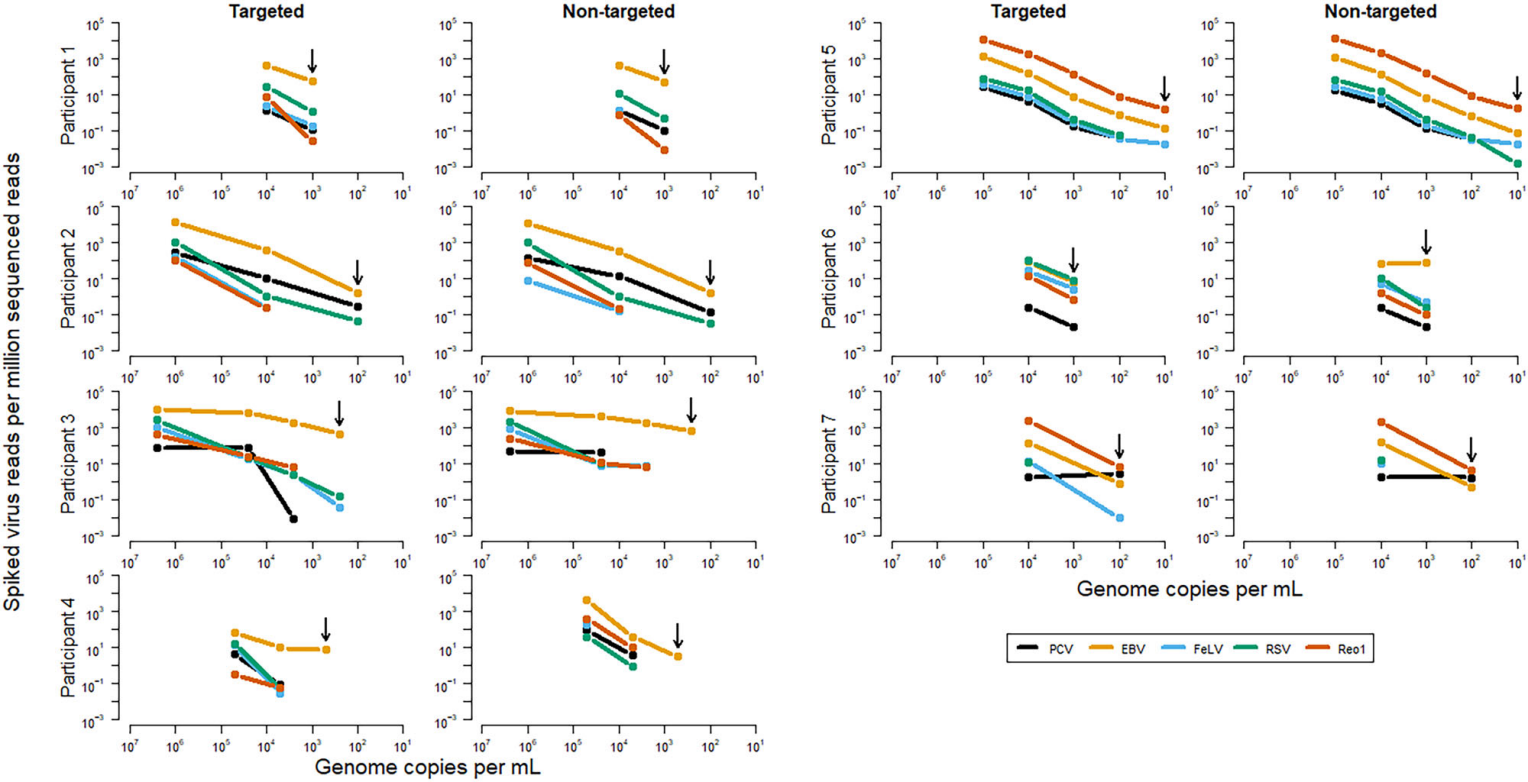
Detection of the five spiked reference viruses by each participating laboratory. Relative abundance (reads per million reads) is shown for each participating laboratory for targeted and non-targeted bioinformatic analysis of reference viruses spiked in the background of 10^9^ adenovirus genomes per mL. EBV, FeLV, RSV, Reo1 and PCV1 were spiked from a range of 1–5 × 10^6^ to 1–5 × 10^1^ GC/mL. Vertical black arrows indicate the last dilution tested by each participant. The colors indicate the different viruses as shown in the figure key. The figure was generated using the R v4.1.1 default plot function.

**Fig. 2:**
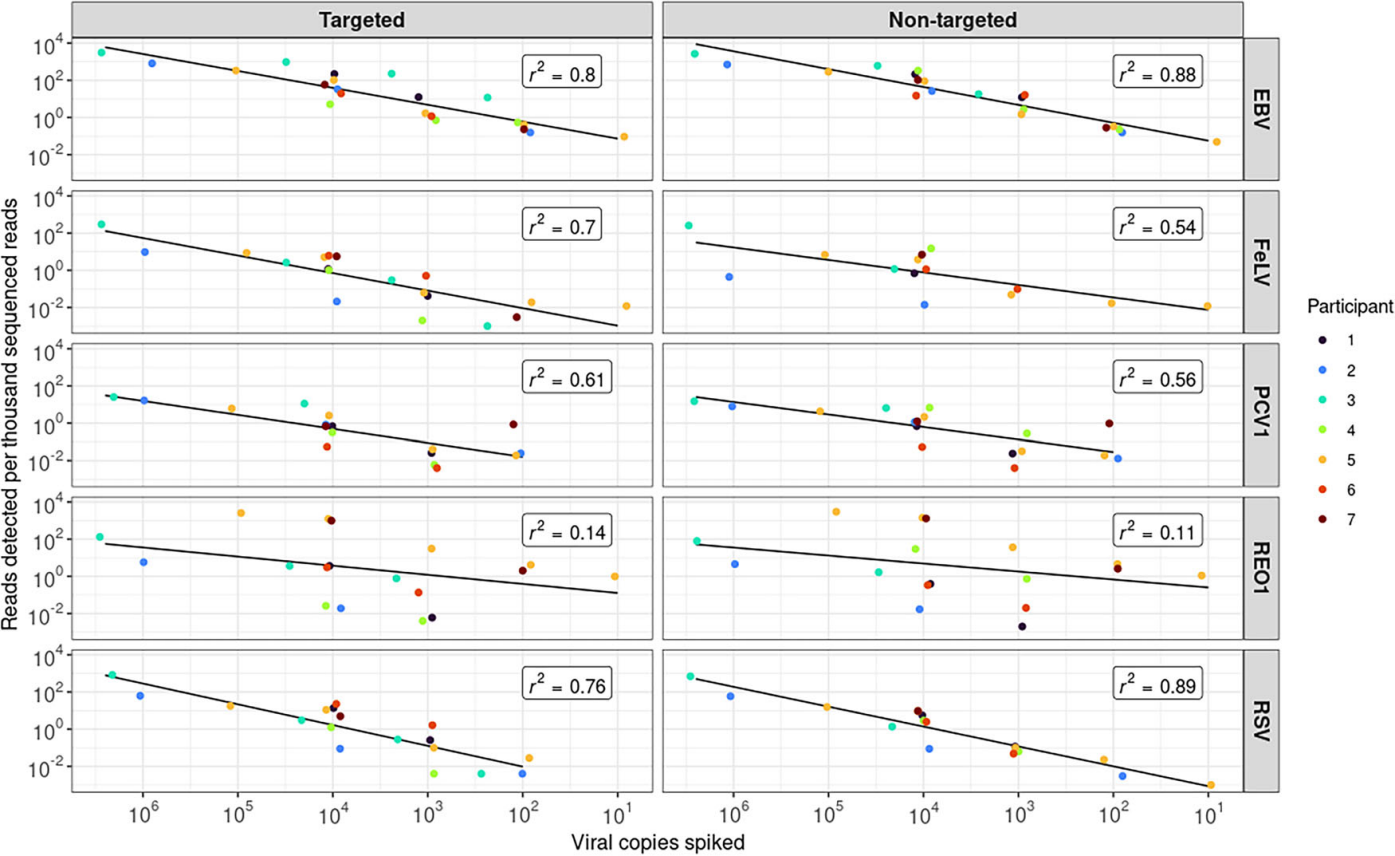
Detection of reference viruses by participating laboratories. Virus detectability represented as a dot plot function of the number of identified viral reads (normalized per thousand sequenced reads) per level of virus spiked into the Ad5 matrix. Individual panels correspond to the virus spiked and targeted and non-targeted bioinformatic analyses are shown in the left and right panels, respectively. Each dot represents an individual participating laboratory in the study: SL-4 was tested by all the laboratories. Linear model fit was generated with the Pearson correlation coefficient calculated to evaluate the inter-laboratory variability. Low r^2^ value reflects high variability. GC per mL is indicated for the starting material. Small data-point jitter (0.1) on the horizontal axis was introduced to allow a distinction of individual datapoints. The plots were generated in R (v. 4.3.1) using the dplyr (v. 1.1.3), ggplot2 (v. 3.4.3), ggpubr (v. 0.6.0) and ggsci (v. 3.0.0) packages.

In addition to assay sensitivity, the assay linearity of the five reference viruses was evaluated to understand the correlation between the relative abundance of mapped reads and the spiked genome copy number in the test samples sharing the same matrix (high titer adenovirus stock). Figures 1 and 2 illustrate the trends for detection of each of the viruses relating to the level of spike, based on the targeted and non-targeted HTS analysis (see Supplementary Table 2 for the read data). It is noted that it was not feasible in this study to perform a sufficient number of replicates in order to assess linearity as only few common spike levels were used by several laboratories,

The results showed the capabilities of HTS for sensitive and broad virus detection even in the absence of linearity, particularly at low titer (Fig. 1). The reduced virus detection for some viruses by certain laboratories was most likely due to the experimental protocols and bioinformatics pipelines used at the time of this study. The overall results showed that the sensitivity of detection of some viruses was affected by the different workflows: this was particularly applicable to Reo1 and PCV1, whereas EBV was least affected. In general, similar results were seen with the targeted and non-targeted analysis suggesting the difference in virus detection was due to protocols upstream of the bioinformatics pipeline.

The detection of the different reference viruses by each of the laboratories is illustrated in Fig. 2. It is noted that the scatter around the trend line was greater for some viruses, especially Reo1, which had a very low r^2^ value. This might be a result of the nature of the dsRNA genome leading to possible difficulties in sample preparation for HTS and suggests an opportunity for optimizing the detection of dsRNA viruses at this step in the workflow. The limited scatter of the other viruses shown in Fig. 2 demonstrates a similar detection level, despite significant differences in the methods used (described in Materials and Methods). However, as shown in Fig. 1, all laboratories did not obtain the same sensitivity for detecting all the spiked reference viruses. This may be due to different bioinformatics criteria and decision trees used for calling a positive signal.

## Discussion

HTS applications for virus detection in environmental and medical research have significantly contributed to increasing the known virome and the resulting successive updates of virus taxonomy^23,24^. HTS analysis of clinical materials has resulted in pathogen discovery^22,23^ and HTS research in biological manufacturing has identified unexpected known viruses such as PCV1 in a licensed rotavirus vaccine and a novel rhabdovirus in the insect *Spodoptera frugiperda* Sf9 cell line, that is a common cell substrate for baculovirus-expressed products^6,7,25,26^. The capabilities of HTS for broad adventitious virus detection has led to the general recognition by regulators, industry, and contract research organizations and service providers for using HTS for replacing the currently recommended in vivo animal assays and PCR assays and supplementing or replacing the in vitro cell culture assays^9–11^. Such a transition is aligned with the global 3 R’s initiative for replacement, reduction, and refinement of animal use^27,28^. The use of HTS can shorten the testing time to accelerate product development and increase product safety by broadening adventitious virus detection to include known and novel viruses. However, there are regional and organization differences in the readiness for using HTS globally, emphasizing the critical need for easily available reference materials, development of standard protocols, and assessment of HTS for analyzing different types of materials involved in the manufacturing of biologics.

To facilitate HTS implementation for adventitious virus testing of biological products, this initial study was designed to evaluate the breadth and sensitivity of virus detection by HTS in a biological sample representing a purified vaccine virus seed or a vector virus preparation, i.e., a low complexity matrix with a reduced cellular content but high-virus titer. The results from this study can provide a basis for further studies in complex matrices (i.e., with a higher content of host cell materials depending on the manufacturing process). Furthermore, the study was designed to test different HTS protocols that would be similar to the real-life situation, since currently there is no common protocol for HTS applications in biologics. To demonstrate the breadth and sensitivity of virus detection, spiking studies were done with five viruses with distinct physical properties (such as large to small range in particle and genome size, with and without an envelope), varying resistance to chemical treatment, and different genome types (such as DNA and RNA, single stranded and double stranded, linear, and circular). These viruses are currently available as CBER NGS Virus Reagents^20,21^ for testing the performance of the different steps in the entire HTS workflow (such as sample processing, cDNA library preparation, sequencing, and bioinformatics pipelines).

The HTS workflow of the individual laboratories represented a wide range of protocols for the upstream and downstream steps for evaluating the analytical sensitivity of short-read HTS for the detection of adventitious viruses. The various protocols used by the different laboratories provided an opportunity to review the steps in the HTS workflow that may or may not be important for optimization to enhance virus detection (Supplementary Table 3). These included pre-treatment steps such as low-speed centrifugation for removal of whole cells and cellular debris (laboratories 2 and 7), ultracentrifugation to separate virus particles from free nucleic acids (laboratory 6), and ribosomal RNA depletion to reduce interference due to residual host cell background (laboratories 1, 6, and 7). We expect that these steps did not have a significant impact since we processed a low complexity sample (with reduced cellular content). The impact of the nucleic acid extraction method using the conventional phenol-chloroform-isoamyl (PCI) protocol (laboratory 3) or silica-based kits (laboratories 1, 2, 4, 5, 6 and 7) could not be evaluated since viral genome copy number recovery was not quantified to determine the impact. Nevertheless, the conventional PCI protocol did not outperform the kit extraction method determined by the detection limit shown in Tables 1 and 2. The library preparation, which included processing of DNA and RNA separately (laboratories 1, and 6) or combined (laboratories 2, 3, 4, 5, and 7), also did not seem to have an impact on the results in such a matrix. Using a one-step RNA-Seq library preparation kit (laboratory 3) or performing the cDNA synthesis step separately prior to DNA-Seq library preparation (laboratories 1, 2, 4, 5, 6, and 7) indicated the latter would be a preferred choice. The benefit of applying low-speed centrifugation and ribosomal RNA depletion could not be concluded in this study by spiking samples with low complexity. However, these pre-processing steps could improve the detection in test materials with a high-complexity background and should be evaluated in further virus spiking studies within different matrices.

Additionally, an unexpected effect of the higher spiked levels of the reference viruses was seen on the sensitivity of detection of the high-titer Ad5 virus in the low-complexity sample used in this study. For the laboratories testing the spiking samples with high titer virus (10^5^ or 10^6^ GC/mL; laboratories 2, 3, and 5), the Ad5-mapped reads were noticeably less than the ones with lower spiking titers in laboratories 2 and 3 (data not shown). This suggests a competition effect resulting from a high copy number of total viral genomes or perhaps due to specific virus genomes of the spiked viruses, which might influence the accuracy of virus detection in such samples. This effect, along with other factors described above should be taken into account when developing and evaluating the HTS protocols for adventitious virus detection.

Different HTS platforms did not seem to have an impact on the results compared as reads per million (NextSeq used by laboratories 1, 2, 5, 6, and 7 and HiSeq used by laboratories 3 and 4), since both were capable of detecting a low level of spiked viruses. These results suggest that the preparation of total nucleic acid using a commercial kit, along with a separate cDNA synthesis step followed by DNA-Seq library preparation using a low input library preparation kit could have contributed to the improved detection of the spiked viruses in this study. Laboratory 3 noted that the poor detection of the PCV1 DNA virus was most likely due to using an RNA-Seq kit for sequencing both DNA and RNA viruses (this step has since been modified). Interestingly, the ultracentrifugation step applied by laboratory 6 for viral particle enrichment, which has been recommended by several metagenomic studies to improve virus detection^29,30^, showed similar results to the other laboratories. Ultracentrifugation might be beneficial for the samples with a complex background such as crude harvest from production, whereas in this study the sample represented a less complex material such as a purified virus seed or vector virus preparation.

The bioinformatic analysis was performed both targeted, to determine the sensitivity of detection towards the predefined reference viruses, and non-targeted using the relevant reference database independently developed by the participants to demonstrate the breadth of virus detection by reporting known and other related sequences or unexpected sequences. The sensitivity of detection for the five viruses was similar in both the targeted and non-targeted analyses (SL-3 and SL-4, respectively); however, a different variability of virus detection was seen by the different laboratories based on their protocols. Sequences that were dissimilar to those of the expected virus genomes (reference viruses and additional viruses) were identified by the non-targeted analysis (Table 3). These included sequences assigned to closely related viruses, either of the same species or within the same genus. These sequences may correspond to sequencing errors that align by chance with sequences of closely related viruses, or to sequences shared within the same taxonomic groups (species, genus, etc.). Most pipelines return one of the “best” hits (heuristic sequence search method) in the list, requiring post-analysis to recognize the closest reliable taxonomic assignment. The second category of non-spiked virus sequences corresponded to other viruses that were present in the host cell used for propagation of the reference virus stocks, endogenous retroviruses, or other viruses for which the source could not be identified. The difference in detection of these sequences by different laboratories can be due to the different bioinformatics pipelines, which may or may not have a follow-up for the removal of false-positive hits based on their criteria. Such sequences can be eliminated based on post-analysis examination based on their presence in negative controls, their ubiquitous distribution in datasets of different samples analysed, and their lack of uniform distribution along the viral genome (stacked reads), among other criteria.

A follow-up study can explore the validity of unexpected signals by cross-analyzing the datasets using individual bioinformatics pipelines and criteria for reporting a positive signal. Since the detection of the spiked-in viruses in a dataset represents the best detection due to the abundance of the reference virus sequence, the additional low-abundance signals detected by different laboratories should be especially informative in this follow-up study, with respect to the detectability of unknowns, including how background noise signal is handled.

## Materials and methods

### Viruses

The five CBER NGS Virus Reagents that were used for spiking in the study are available to support HTS studies from the NIAID BEI Resources, catalogue number NR-59622^21^. They were selected to demonstrate broad virus detection by HTS, based on distinct physicochemical and genome properties of the viruses. These viruses were previously described and established as WHO International Reference Reagents^31^, but were discontinued due to their replacement with a new reference panel of seven viruses, established as the 1^st^ WHO International Reference Panel for Adventitious Virus Detection by HTS^20^. The general properties are shown in Table 4.

**Table 4:** Properties of viruses used for spiking study

Virus/Gene	Topology	GC per mL^a^	Virus genome length (bp)	GenBank accession number
Reo1/ L1	RNA, double-strand; linear (segmented), non-enveloped	2.4 × 10^9^	3854	M24734.1
Reo1/ L2			3915	AF378003.1
Reo1/ L3			3901	AF129820.1
Reo1/ M1			2304	AF461682.1
Reo1/ M2			2203	AF490617.1
Reo1/ M3			2241	AF174382.1
Reo1/ S1			1458	M10260.1
Reo1/ S2			1331	L19774.1
Reo1/ S3			1198	M18389.1
Reo1/ S4			1196	X61586.1
RSV	RNA; single-strand; linear, enveloped	1.04 × 10^9^	15,158	JF920069.1
FeLV	RNA; single-strand; linear (dimeric), enveloped	5.3 × 10^10^	8448	NC_001940.1
EBV	DNA, double-strand; Linear, enveloped	3.7 × 10^8^	172,281	V01555.2
PCV1	DNA, single-strand; circular, non-enveloped	2.7 × 10^11^	1758	NC_001792.2

^a^Based on ddPCR assay results provided in the Certificate of Analysis from the American Type Culture Collection (ATCC, Herndon, VA).

It should be noted that EBV and PCV1 also contained additional viruses due to the cell lines used for their production. The GC/mL of SMRV in the EBV virus stock was determined to be 3.0 × 10^9^ and PERV in the PCV1 virus stock was determined to be 1.4 × 10^9^ by laboratory 3 Reference genome used for SMRV analysis was GenBank number NC_001514.1 (SMRV-H). Reference genome for PERV analysis was GenBank accession number AF038600.1 or PERV-A KY484771.1. Additionally, the presence of *ungulate copiparvovirus 5* (bovine serum associated, bovine bosavirus) was confirmed in this study in PCV1, EBV, and Reo1.

Ad5 was obtained from ATCC; Cat# VR-1516) and genome copy number determined by laboratory 5 using ddPCR assay (3.35 × 10^11^ GC/mL). Reference genome used for Ad5 analysis was GenBank accession number AY339865.1.

### Study design

Eight laboratories initially participated to evaluate LOD of the five references viruses using HTS Illumina short-read platforms. Each laboratory used the same genome copy number of each of the viruses to spike at different levels into a high titer (10^9^) Ad5 matrix. Laboratory 6 added Ramos cell DNA to increase the complexity of the background. All the laboratories tested 10^4^ genome copies of the mixed virus spiked in 10^9^ Ad5 and some laboratories included other spiking levels (ranging from 10^6^ to 10^1^ genome copies, SL-6 to SL-1, respectively). Each laboratory used their own HTS workflow from sample preparation (including any pre-treatment steps) through the bioinformatics pipelines. Virus detection included targeted and non-targeted analysis. One laboratory could only participate in the 10^4^ genome copy spiking level with targeted analysis, which was submitted along with the other seven laboratories to support the establishment of the earlier WHO International Reference Reagents^32^ and not included in this study. For simplicity, the technical and bioinformatics details of the steps in the HTS workflow have been presented by laboratories 1 - 7 in the Supplementary Table 3 and are described briefly for each laboratory in the next section. The raw data analysis is shown in the excel file in Supplemental Table 1. The raw sequencing read files are available in NCBI under BioProject PRJNA1011899.

### High-throughput sequencing

Independent workflows were used by each of the laboratories from sample processing through bioinformatics. All laboratories used different platforms of the short-read Illumina technology.

### Laboratory 1

Spiked samples were generated by adding the intended genome copies of each virus followed by dilution up to 1 mL with media. For DNA extractions, 200 µL was extracted using the QIAamp MinElute Virus Spin kit (Qiagen, Cat. No. 57704) according to the Manufacturer’s Protocol with an elution volume of 30 µL of which 11.5 µL was used for cDNA synthesis according to the manufacturer’s protocol for the SuperScript Double-Stranded cDNA Synthesis Kit (Invitrogen, Cat. No. 11917010). The final sample was concentrated with MinElute PCR clean up kit (Qiagen, Cat. No. 28004) to a final volume of 15 µL. Libraries were prepared with 13 µL cDNA using the Nextera DNA Flex kit (Illumina, Cat. No.20060060) according to the manufacturer’s protocols in a final volume of 30 μL and library was quantified using Qubit dsDNA HS Assay Kit (Thermo Fisher Scientific, Cat. No. Q32851). Libraries were denatured to 4 nM per Illumina manufacturer’s protocol prior to being loaded and run on NextSeq 500 (NextSeq 500/550 High Output Kit v2.5 (300 Cycles; Illumina, Cat. No. 20024908) in a paired-end mode. The read length was set to 2 × 150 bp.

For RNA extractions, 140 µL was extracted using QIAamp Viral RNA Mini Kit according to the manufacturer’s protocol (Qiagen, Cat. No. 52904) with an elution volume of 30 µL of which 10 µL was used according to manufacturer’s protocol in the Hyper KapaPrep with RiboErase for RNA prep (Roche, Cat. No. KK8560) and quantified library using Qubit dsDNA HS Assay Kit (Thermo Fisher Scientific, Cat. No. Q32851). Libraries were denatured and run as described above for the DNA sequencing except the read length was set to 2 × 75 bp.

For bioinformatic analysis, data was converted from the Illumina BCL image format to FASTQ sequence quality-scoring format using BCL2FASTQ v2.20. Quality assurance of decompressed FASTQ files was performed using FastQC v0.11.3^33^. Subsequent quality control of the FASTQs was performed by use of Trimmomatic v0.36^34^. Alignment was first performed against the Chinese hamster ovary (CHO) K1 cell line reference genome (NCBI Accession: GCA_003668045.2) assembled by Xu et al.^35^ using Bowtie2 v2.2.1^36^ and Samtools v1.9^37^ as part of the traditional testing pipeline, even though it is not as relevant to this study.

For targeted analysis, unmapped reads were aligned against custom database only containing the viral target sequences known to be present in the spiked samples, then output into plain text sequence alignment mapping (SAM) format. Genomic breadth-of-coverage values were subsequently computed using BEDtools v2.27.2, and a tab-delimited table of relationally joined results was generated using custom tooling to generate the final report. For non-targeted analysis, unmapped reads were aligned against full-length sequences from RVDB v19.0^15^. All remaining unmapped reads after this alignment were aligned again to the remaining sequences in the RVDB v19.0 before all RVDB-related alignments are merged using Samtools and then output into plaintext SAM format. Genomic breadth-of-coverage values were subsequently computed using BEDtools v2.27.1^38^, and a tab-delimited table of relationally joined results was generated using custom tooling. Filters were implemented to remove “background noise” at the end of the pipeline. The final report was generated and reviewed by a virologist. “Hits” were individually confirmed or eliminated using BLAST searches, taxonomy, and sequence alignment pileups.

### Laboratory 2

The Ad5 matrix was diluted to 10^9^ GC/mL, which was prepared prior to any work with a panel of the five viruses at either 10^2^, 10^4^ or 10^6^ GC/mL. Controls included ddH_2_O spiked with the five viruses at 10^4^ GC/mL and the Ad5 matrix at 10^9^ GC/mL with the spiked viruses.

Following the spiking of the viruses into the Ad5 matrix the nucleic acid was extracted from the entire sample (1 mL) using a QIAamp MinElute Virus Kit (Cat. No. 57704) and entirety of eluate converted into the cDNA by SuperScript IV VILO Master Mix (Thermo Fischer Scientific, Cat. No. 11756050). The cDNA was then fluometrically assessed for dsDNA content using a Quant-iT™ PicoGreen™ dsDNA Assay (Thermo Fischer Scientific, Cat. No. P11496).

For the library preparation 1 ng of each sample (102 µL) was used with Nextera XT DNA Library Preparation Kit (Illumina) according to the manufacturer’s instructions. Following the library preparation, the libraries were quantified using a KAPA qPCR (Roche, Cat. No. KK4824) method.

Libraries were sequenced on NextSeq 550 (NextSeq 500/550 High Output Kit v2.5 (300 Cycles), Illumina) in a paired end mode. The read length was set to 2 × 151 bp.

For bioinformatic analysis, raw paired-end reads were first assessed for quality as well as length and subsequently trimmed. High-quality reads were those with a Phred quality score of 20 over 75% of bases and a minimum length of 50 bp. High-quality paired-end reads were treated as single-end sequences and mapped by means of BWA-MEM to a database index of 2,643,953 viral entries (non-targeted approach) or a database of viral spike genomes (targeted approach). The viral database consists of viral genome sequences from Genbank and RefSeq augmented with entries from RVDB version 20. Reads that mapped to viral entities were extracted and subsequently mapped to the index of the nr/nt database (non-redundant nucleotide) for conformation of the sequencing read source. Reads that generated an equal or higher mapping score to a viral database entry than an NT database sequence were considered for downstream analyses. Subsequently, “hits” were determined by the alignment of sequencing reads to a viral database sequence. Specifically, a “hit” should be at least 100 base pairs in length, exhibit a similarity of at least 90%, and demonstrate specificity to a particular virus sequence or family. Viral genome sequences with at least one “hit” were reported. However, reads that mapped to non-specific genomic locations (e.g. poly-T repeats) were not considered true positive hits and therefore did not contribute to reporting of results.

### Laboratory 3

Samples (1 mL) were prepared in DMEM medium containing the five viruses at 10^6^, 10^4^, 10^3^, and 10^2^ genome copies in 10^9^ genome copies of Ad5, along with Ad5 without spiked viruses as background control and DMEM-only as no-template control (NTC).

Total nucleic acid was extracted by the phenol/chloroform method. Briefly, 400 µL sample was mixed with the digestion buffer containing 10 mM Tris-HCl, pH 7.5 (Quality Biological, Cat. No. 351-006-721), 125 mM NaCl (Invitrogen, Cat. No. AM9759) and 0.625% SDS (Quality Biological, Cat. No. 351-032-721). The mixture (final volume 500 µL) was digested by 65.92 µg proteinase K (Roche, Cat. No. 03115828001) at 56 °C for 3 hours followed by extraction with phenol/chloroform/IAA (25:24:1, Invitrogen, Cat. No. 15593-031) and ethanol precipitation.

After washing with 70% ethanol, the pellet was resuspended in 50 µL nuclease-free water (Ambion, Cat. No. AM9937).

Seven microliters of the total nucleic acid extraction were taken for library preparation. The sequencing libraries were generated by SMART-Seq Stranded Kit (Takara Bio; Cat. No. 634442) by following manufacture’s protocol except the ribosomal RNA depletion was not performed. The quality and quantification of libraries were assayed by Agilent Bioanalyzer 2100.

Sequencing was performed by Illumina HiSeq 2500 sequencer with paired-end, 101 bps sequencing mode. Six sequencing runs were carried out individually for each of the six samples (four spiked and two controls). The first three bases of reads were trimmed by Cutadapt^39^.

For targeted analysis, the raw FASTQ reads for virus spiking-in stocks generated from Illumina HiSeq-2500 platform with paired-end (2 × 101 bp) mode were processed by CLC genomic workbench version 12.0.1 (Qiagen). Unless mentioned, the modules were applied with the default settings. The raw reads were trimmed to remove the sequencing adapter and low-quality reads by the Trim Reads module, followed by mapped to the reference viral genome for each spiking virus by using Map Reads to Reference module. The mapped tracks were subjected to QC for Read Mapping module to obtain the information of the coverage and sequencing depth for each virus. Targeted-mapped reads were subjected to BLASTn analysis for counter-screening. The positive call was made only if the read was indicated to the closely-related taxonomies under family level.

For non-targeted analysis, the paired-end raw reads were trimmed by BBtools BBduk v38.69 package (40) to remove the low-quality reads [quality (Q) score is less than 30, the length is less than 50 bp and the reads contained known Illumina sequencing adapters or vectors]. The pre-trimmed, raw paired reads and trimmed ones were subjected to FastQC v0.11.8 to ensure the trimming effectiveness confirmed by the improvement of quality matrices after trimming. The trimmed paired reads were merged to a single, interleaved file by BBduk reformat v38.69 package^40^. The merged reads were collapsed by CD-HIT-EST v4.8.1^41^ to one representative if sharing 100% similarity with other reads. The collapsed reads were aligned against U-RVDB Version 20.0 by BLASTn 2.10.1 + ^42^. The BLASTn hits were collapsed by unique organism name while the organisms with highest hits number were retrieved. The trimmed, non-collapsed paired reads were aligned to the genome collection of retrieved organisms by Bowtie2 v2.4.4^36^, and the overall coverage information was obtained by samtools coverage v1.12 package^37^. Meanwhile, the NTC mapping result was used to mask the alignment collection index by bedtools bamtobed v2.29.0 package^38^ to avoid false-positive hits. Hits were removed if the mapped length was less than 50 bp and the organisms were eukaryotes. The counter-screen was performed to validate the positive calls. The reads resulting in positive calls were collected and aligned to NCBI NT by BLASTn 2.10.1 + ^42^. The positive calls were verified if the output hit from RVDB BLAST and NT BLAST agreed with each other in the species level. For the calls with less than 40% of the alignment length, they were subjected to secondary follow-up to ensure the aligned loci was not with low complexity.

### Laboratory 4

Total nucleic acid was extracted from all samples (200 µL starting material) with the QIAamp Cador Pathogen Mini Kit (Qiagen, Cat. No. 54104) according to the manufacturer’s recommendations, except the application of carrier RNA. Final elution volume was 100 µl. Single stranded DNA and RNA was converted to cDNA by using the Invitrogen SuperScript III First-Strand Synthesis System for RT-PCR Kit (Cat. No. 18080-051, lot# 1844575) and QIAGEN QuantiTect Whole Transcriptome Kit (Cat. No. 207043, lot# 154053166) with 7 µL input volume. Libraries were then prepared using the Illumina TruSeq Nano DNA Library Prep Kit (Illumina, Cat. No.20015964) according to the manufacturer’s recommendations. Finished libraries were sequenced with the Illumina HiSeq 2500 Sequencer system in a 2 × 100 paired-end read mode. For targeted analysis, sequences were adapter trimmed with fastp^43^ by using default settings. Reads were then mapped against the reference sequences by using BWA-MEM (v0.7.12). Data formatting was done with Samtools (v0.6.8)^37^ and Picard (v0.7.17)^44^. Subsequently, depth of coverage was calculated by using the gatk toolkit^45^.

For non-targeted analysis, raw sequencing data is processed with fastp to remove poor quality bases (below Phred Quality 20) by employing a sliding window approach. After quality trimming, adapter sequences in the reads are removed. Further, shorter reads which are < 30 bp length are also removed to retain only high-quality sequencing reads for each sample in the analysis. In case of paired-end reads, both the sequencing reads which pass the QC criteria are considered for downstream analysis.

The first pipeline step after quality cleaning is a host filtering step. High-quality reads are further processed using KrakenUniq^46^ to detect and remove reads that could be assigned to a host genome (human, mouse, rat etc.). The host filtering step was deactivated for sample analysis. Adenovirus host filtering was not applied, as it is within the scope of viral detection (see below).

The second noise reduction step is a non-virus read removal step (“microbial contaminants”, like bacteria, archaea, fungi, protozoa). Only complete archaeal genomes published by NCBI were included. If a microbial contaminant was found which has no representation in NCBI’s complete genome database, the nearest neighbor was determined using a lowest common ancestor algorithm to classify at a higher taxonomic level.

After the noise reduction steps, highly covered virus genomes were detected. For this purpose, the remaining reads after sequence cleaning and filtering were aligned to a curated high-quality virus reference database (clustered RVDB v20^15^) using BWA-MEM^47^ and subsequent genome coverage analysis with BEDtools^38^. The approach allows for viral variants via softclipping within the mapping algorithm. In this approach a 10% coverage breadth threshold was included. Additionally, all sequence entries below 750 bp in the reference database were excluded. These cutoffs were implemented to avoid false positive results because of low complexity regions and incomplete fragmented sequence database entries.

The pipeline includes a representative genome strategy. The first step of the process is read mapping versus a reference database. All hits are taxonomically classified. The hits to all viruses are collected and filtered with a coverage breadth cutoff to find relevant hits. For all virus species above the cutoff, all strains with hits within this species are collected (according to the taxonomical information). From all of these strains a representative genome (aka “top accession”) is chosen (according to completeness (largest size) and coverage) and all reads mapped to all strains within the species are mapped again versus the representative genome to determine the final read numbers and coverage.

Finally, based on the virus reference database results, coverage, taxonomy and manual results inspections (follow-up) virus reads are classified, quantified and reported.

### Laboratory 5

Total nucleic acid was extracted through five parallel extractions (5 × 200 µL starting material) from all samples with the Qiagen MinElute Virus Spin Kit (Qiagen, Cat. No. 57704). Final elution volume was 20 µL for each extraction. Elution from each extraction (5 × 20 µL) were pooled together and concentrated using the Qiagen MinElute PCR Purification Kit (Qiagen, Cat. No. 28004) with an elution volume of 12 µL of which 10 µL were used for cDNA synthesis according to the manufacturer’s protocol for the SuperScript Double-Stranded cDNA Synthesis Kit (Invitrogen, Cat. No. 11917010). The final sample was concentrated with MinElute PCR Purification Kit (Qiagen, Cat. No. 28004) to a final volume of 10 µL.

Sequencing libraries were prepared from double-stranded DNA by tagmentation using the Nextera XT DNA Library Prep Kit (96 samples) (Illumina, Cat. No. FC-131-1096). The sequencing of each sample was performed in a single run of an Illumina NextSeq instrument (2 × 150 paired-end sequencing). A total of six sequencing runs were carried out (one run for each spiking level and one run gathering negative controls). Controls included: 1 mL of TE present 1x during nucleic extraction and added to the workflow at the cDNA synthesis step and 200 µL of TE 1x that went through the workflow from the nucleic acid extraction. Each control was analyzed twice.

Raw sequencing reads were first demultiplexed using BCL2FASTQ v2.17.1.14 to extract all reads pertaining to each sample. Quality of paired-end sequencing reads was first checked using the FastQC v0.11.5 software^48^. Next, trimming of adapter/index sequences and trimming of low-quality bases was achieved using Trimmomatic v0.36^34^. Of note, minimum read length of 50 bp and 25 bp were used respectively for targeted and non-targeted analyses. After this stage, all remaining reads were considered high-quality reads and ready to be analyzed.

For the targeted analysis, reads associated to each sample were processed using an internal data analysis pipeline for targeted adventitious virus detection. High-quality reads were aligned using BWA v0.7.12^47^ to a custom sequence database containing NCBI reference sequences for all viruses expected to be present in the samples: Ad5 (GenBank: AY339865.1), PCV1 (GenBank: NC_001792.2), EBV (GenBank: V01555.2), FeLV (GenBank: NC_001940.1), RSV (GenBank: JF920069.1), Reo1 (GenBank: M24734.1, AF378003.1, AF129820.1, AF461682.1, AF490617.1, AF174382.1, M10260.1, L19774.1, M18389.1, X61586.1), PERV (GenBank: AF038600.1) and SMRV (GenBank: NC_001514.1).

Samtools v0.1.18^37^ combined with internal scripts was used to extract essential statistics such as number of mapped reads per virus and coverage. The percentage of identity was derived from the number of variants discovered by BCFtools v1.3.1^49^.

For non-targeted analysis reads associated to each sample were processed using an internal data analysis pipeline for adventitious virus detection based on considerations for optimization of high-throughput sequencing bioinformatics pipelines for virus detection^14^.

High-quality reads were aligned to the Ad5 reference genome (GenBank: AY339865.1) considered as host in this analysis, using BWA v0.7.12^47^. The reads that did not map to the Adenovirus 5 genome were extracted using Samtools v0.1.18^37^ and then aligned, using BWA, to the reference virus database, RVDB v18.0^15^, to screen for potential virus reads.

During counter-screening^14^, reads aligned to RVDB were extracted and then aligned to a subset of GenBank v236 sequences using BLAST v2.10.0 + ^50^ to assess their origin. The subset of GenBank excluded divisions containing constructed sequence entries (CON), expressed sequence tag entries (EST), genome survey sequence entries (GSS), high throughput genomic sequencing entries (HTG) and transcriptome shotgun assembly sequence entries (TSA) that are redundant with other divisions, e.g., gene fragments or genome fragments. In an approach similar to TAMER^51^, BLAST results were analyzed in a mixture model to estimate the proportion of reads generated by each candidate genome or sequence. The result of the counter-screening is a taxonomical assignment of the reads consisting mainly in a table with the GenBank accessions of sequences considered as present in the sample tested, their description, their taxonomy, the number of reads mapped to those accessions and the length coverage. Reads assigned to each accession sequence were extracted and realigned only to the assigned accession sequence. Depth of coverage graphs presenting the number of reads covering each nucleotide of reference sequences are then automatically computed for each accession from alignment files.

Virus species were considered as having a high likelihood of presence in the samples if ≥ 10 reads were observed for a species and ≥ 300 nucleotides were covered on the species accession sequence capturing the highest number of reads.

Results for viruses that were not bacteriophages nor virophages were reported in Supplementary Table 2.

### Laboratory 6

Samples were diluted in nuclease-free water and the model adventitious viruses (RSV, FeLV, Reo1, EBV and PCV1) were spiked in at 10^4^ and 10^3^ GC/mL in high copy number Ad5 (10^9^ GC/mL). Lysates of Ramos cells (3 × 10^6^ cells) were obtained by three freeze-thaw cycles and added to the mix of Ad5 with the spiked viruses with the objective to get a concentration of nucleic acids following ultracentrifugation sufficient for the construction of libraries. Ad5 spiked at 10^9^ GC/mL in nuclease-free water and unspiked lysates of Ramos cells were run in parallel with the spiked samples.

The spiked samples were ultracentrifuged at 110,000 × g for 3 h at 4 °C on a 20% sucrose cushion to concentrate viral particles. The pellet was then rehydrated overnight at 4 °C with 200 µL of PBS and resuspended. The 200 µL of ultracentrifuged samples were submitted to nucleic acids extraction using the Indispin Pathogen Mini kit (Indical; Cat. No. SP54104).

To increase the capacity of adventitious virus detection, extracts were split in two aliquots of 25 µL and one aliquot was submitted to a DNase treatment using Baseline-zero DNAse (Euromedex; Cat. No. DB0715K), the second aliquot was submitted to a RNase treatment using RNAse A (ThermoFisher Scientific, Cat. No. EN0531).

DNA and RNA quantification were assessed using the Qubit assay kits (ThermoFisher Scientific, Cat. No. ARN:Q32852 and Q10210; ADN: Q32854), the quality of the extract was checked using the Nanodrop instrument.

DNA libraries were prepared from DNA extracts, using the xGen ssDNA & Low-Input DNA Prep kit (Integrated DNA Technologies; Cat. No. 10009817). The quantification and qualification of the libraries were performed using the Qubit instrument and the Bioanalyzer 2100. RNA-Seq libraries were prepared from RNA extracts, using the SMARTer Stranded Total RNA-Seq kit Pico Input (Ozyme; Cat. No. 635005/635006/635007). The process includes an rRNA depletion step and the addition of a tag allowing the identification of the template strand used for RNA transcription. The quantification and qualification of the libraries were performed using the Qubit instrument and the Bioanalyzer 2100.

Sequencing was carried out on the Illumina NextSeq500 instrument using the NextSeq 500/550 High Output kit v2.5 (150 cycles). Sequencing was single-read and read length was of 150 nucleotides. Sequence data were converted from the Bcl to FASTQ formats using the Illumina BCL2FASTQ software (v2.17.1.14).

For targeted analysis, the raw data reads were filtered to select high-quality and relevant reads. Raw data was sorted to suppress or cut duplicates (PCR/optical), low quality reads (Q ≤20) and homopolymers. Sequences introduced during the preparation of Illumina libraries (adapters, primers) were removed with Skewer^52^. Reads mapped with BWA^47^ onto i) the human genome [Reference GRCh37/hg19^53^] used as host background, or ii) bacterial rRNA, were discarded. The bacterial rRNA database was initially downloaded from the EMBL-EBI ENA rRNA database (ftp://ftp.ebi.ac.uk/pub/databases/ena/rRNA/release) and reworked using in-house sequence cleaning and clustering processes. Remaining reads were considered as sequences of interest and used for subsequent analyses.

For mapping on reference genomes, reference viral genomes were first indexed enabling high-quality reads to be mapped on those references via BWA (mem module with T parameter set to 60). An estimate of viral genome coverages was obtained using BWA sam outputs as inputs for Samtools (reads sorting) and BEDtools (genomecov module).

For non-targeted analysis, the raw reads were filtered to select high-quality reads. Duplicate, low-quality and containing homopolymers reads (*r* = 0.9*l with l being length of read) were removed while those still including adapters were trimmed using skewer software with default parameters^52^, too short reads after trimming (*l* ≤*60* nucleotide) being removed. Genetic background of the host was removed by mapping the host genome and traces of environmental bacteria were eliminated by screening reads against a comprehensive rRNA database (release available at ftp://ftp.ebi.ac.uk/pub/databases/rrna) using BWA in both cases^47^. Remaining reads, considered as sequences of interest, were then assembled into contigs using Megahit^53^ retaining contigs longer than 100 bp. The resulting contigs and non-assembled reads (singletons) were first sieved using BLASTn^42^ against an in-house viral nucleotide database. In case of any significant hit in the virus only database, it was aligned on a comprehensive nucleotide database. While BLASTn was used to screen contigs against the comprehensive database, Magic-Blast^54^ was used to screen singletons both with *e-value* set to 10^-3^. If the best hit was still a viral taxonomy, hits were reported. Contigs with no viral nucleotide hits were similarly aligned successively on viral and comprehensive protein databases to check for more distant viral hits. The taxonomic assignation reported the best hits with contigs not assigned after these two rounds of alignment being classified as unknown or non-viral species.

Nucleotide viral and comprehensive databases were downloaded from the EMBL-EBI nucleotide sequence database (https://www.ebi.ac.uk/; release.143) and processed by a proprietary software to remove duplicate and low confidence sequences. Protein viral and comprehensive databases were downloaded from the Uniref100 database (https://www.uniprot.org; release 2020_03) and further cleaned. More precisely, an altered entry (i.e., lacking Identifier / common Taxid / Division or gathering too many sequences) was considered doubtful and discarded.

At the end of the process, the non-targeted pipeline provides a comprehensive pdf report summarizing the analysis metrics (filtering, assembly) along with detailed taxonomic tables. Depending on alignments scores (identity percentage, match length), identified viral species were classified into three categories: “close to known taxonomies”, “distant to known taxonomies” or “background noise”. Reads “close to known taxonomies” were reported.

### Laboratory 7

Total nucleic acid (RNA and DNA) was extracted from 900 µL (450 µL for PureLink extraction and 450 µL for Wako extraction) of the starting sample using a PureLink Viral RNA/DNA Kit (Invitrogen; Cat. No. 12280-050) and a DNA Extractor kit (Wako; Cat. No. 295-50201) following the manufacturer’s protocols with some modifications^55^. Ribosomal RNA depletion was then performed on the extracted nucleic acids using the Low Input Ribominus Eukaryote System v2 (Thermofisher; Cat. No. A15027) following the manufacturer’s protocol. Following this step, cDNA synthesis was performed following the manufacturer’s protocol using the SuperScript® Double-Stranded cDNA Synthesis kit from Invitrogen (Cat. No. 11917-010), and an Illumina Nextera XT library was made using the Nextera XT DNA Sample Preparation Kit (Cat. No. FC-131-1024) and the Nextera XT DNA Index Kit (Cat. No. FC-131-2001, FC-131-2002, FC-131-2003, FC-131-2004). The sequencing of each sample was performed using the Illumina NextSeq 500 (2 × 151 bp paired-end sequencing).

The following samples were prepared for this study in a total volume of 1 mL: 10^4^ GC of the reference viruses spiked into 10^9^ GC of Ad5, 10^2^ GC of the reference viruses spiked into 10^9^ GC of Ad5, and nuclease-free water was included as negative control. Multiplexing of the 10^4^ GC of reference viruses spiked into 10^9^ GC of Ad5 and the negative control was done for sequencing of these two samples. The sample containing 10^2^ GC of the reference viruses spiked into 10^9^ GC of Ad5 was not multiplexed.

For the targeted analysis, raw reads were first filtered to remove low-quality entries (whose mean composite Phred score was below Q30) and low-complexity reads [whose mean 64 nt-windowed SDUST complexity was below 2.0^56^]. Then, for each virus used in the study, the reads were mapped to the corresponding reference genome by using NCBI blastn with e-value of 1.0e-5, minimum read coverage of 80% and minimum identity of 80%. The resulting coordinates for the set of matches against the target genome were then converted to SAM format. The SAM file was processed with mpileup (part of the SAMtools suite). The mpileup output was then converted into frequency tables by tallying nucleotide counts and indels. Subsequently, consensus sequences were generated and aligned using MAFFT.

For the non-targeted analysis, raw reads were first filtered to remove low-quality reads (whose mean composite Phred score was below Q20) and low-complexity reads [whose mean 64 nt-windowed SDUST complexity was below 2.0^56^]. Then, reads matching Human mastadenovirus C (from RefSeq NC_001405) were screened out using megablast at 1.0e-25. Reads were trimmed of any 5’ or 3’ N characters and rejected if the trimmed read length was less than 120 nucleotides (80% of our nominal read length of 151 nucleotides). Finally, reads were screened against a custom database of known host sequences (typically absent from RefSeq host assemblies) and were rejected if they matched via blastn over 95% or more of the alignment and at 1.0e-25 or better.

Following these QC and prescreen steps, the remaining sequences were compared against a curated reference genome collection consisting of RefSeq viruses, as well as select GenBank viruses, using blastn at 1.0e-5. Those sequences that matched viruses were then counterscreened against archaea, bacteria, fungi, protists, and selected plant and metazoan species, all from RefSeq, and again using blastn at 1.0e-5. A proprietary algorithm was then used to assign each sequence to its best-fitting taxonomic bin, leveraging both these blastn results as well as phylogenomic information relating the reference genomes to one another.

Sequences determined to be from viruses were then screened more exhaustively against NCBI’s non-redundant nucleotide database, again using blastn at 1.0e-5. Here, though, only matches whose alignment covered 80% or more of the read and with sequence identity of 60% or better were retained. Also, matches whose bit score was less than 95% of the best match were discarded. Sequences meeting these match QC criteria were then compared against the RVDB using tblastx at 1.0e-10 and with the “-seg” option set to “yes”, and against the proteic version of the RVDB using blastx at 1.0e-5, word_size of 3, gapopen of 11 and gapextend of 1. To be reported, these protein matches needed to show at least 60% sequence similarity.

Given this set of confirmed matches to genomes and to proteomes, a decision tree was then applied: Matches needed to be to metazoan viruses, with a minimum of three unique reads mapping to the viral genome, covering at least 301 nucleoties (twice the nominal read length minus 1, given that paired-end sequencing was used). For a hit to be reported, its observed genomic coverage multiplied by its mean sequence identity had to be at least 25% of the expected Lander-Waterman coverage^57^ based on the number of unique reads. Genomic hits required at least one match at the protein level as well.

Signals that passed these criteria are described in this paper, together with any supplementary information retrieved from the NCBI web site in order to conform with what other groups reported, such as taxonomic information. Manual analysis of the data reported was done, and the results were categorized into known spiked viruses, known additional viruses and other viruses. The top accession number for each of the five viral spikes was reported based on number of reads and percent coverage. The taxon ID from NCBI as well as the family, genus and species were also reported for each top accession number for each category of viruses. Phages were not included in the reported results.

## Supplementary information

Supplementary Table 1

Supplementary Table 2

Supplementary Table 3

## Data Availability

The sequence data used to generate the figures present in this manuscript are available from NCBI BioProject PRJNA1011899.
